# Human Chorionic Gonadotropin-Mediated Immune Responses That Facilitate Embryo Implantation and Placentation

**DOI:** 10.3389/fimmu.2019.02896

**Published:** 2019-12-10

**Authors:** Anne Schumacher, Ana C. Zenclussen

**Affiliations:** Experimental Obstetrics and Gynecology, Medical Faculty, Otto-von-Guericke University Magdeburg, Magdeburg, Germany

**Keywords:** human chorionic gonadotropin, uterine immune cells, embryo implantation, placentation, fetal tolerance, pregnancy

## Abstract

Human chorionic gonadotropin (hCG) serves as one of the first signals provided by the embryo to the mother. Exactly at the time when the first step of the implantation process is initiated and the blastocyst adheres to the maternal endometrium, the embryonic tissue starts to actively secrete hCG. Shortly thereafter, the hormone can be detected in the maternal circulation where its concentration steadily increases throughout early pregnancy as it is continuously released by the forming placenta. Accumulating evidence underlines the critical function of hCG for embryo implantation and placentation. hCG not only regulates biological aspects of these early pregnancy events but also supports maternal immune cells in their function as helpers in the establishment of an adequate embryo-endometrial relationship. In view of its early presence in the maternal circulation, hCG has the potential to influence both local uterine immune cell populations as well as peripheral ones. The current review aims to summarize recent literature on the participation of innate and adaptive immune cells in embryo implantation and placentation with a specific focus on their regulation by hCG.

## Introduction

**Figure 1:**
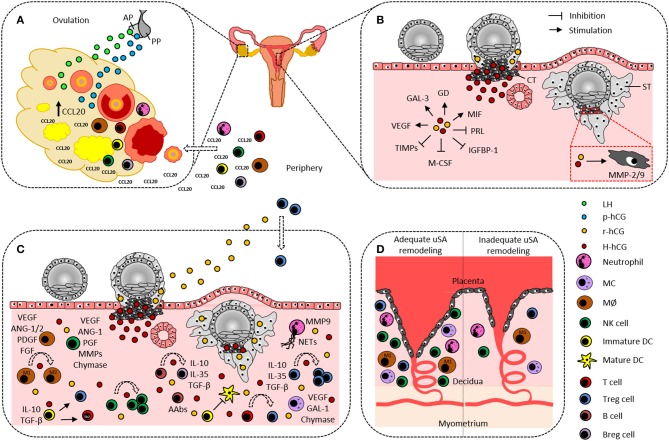
Hypothetical scenario on the participation of human chorionic gonadotropin and immune cells in ovulation and embryo implantation. **(A)** Pituitary gland-produced LH and p-hCG induce ovarian CCL20 secretion that in turn promotes leukocyte influx from the periphery into the ovary. Ovarian DCs and MØ are suggested to actively contribute to the ovulation process. **(B)** Cytotrophoblast-derived H-hCG and syncytiotrophoblast-derived r-hCG promote angiogenesis, trophoblast invasion, and tissue-remodeling by inducing endometrial expression of GAL-3, GD, MIF, and VEGF as well as trophoblastic expression of MMP-2 and−9, and by inhibiting endometrial expression of PRL, IGFBP-1, M-CSF, and TIMPs. **(C)** Decidual innate and adaptive immune cells support embryo implantation by producing and secreting a variety of factors that are indicated aside each immune cell type and are partially induced by hCG. Additionally, hCG confers fetal tolerance by enhancing uterine NK cells, M2 cells, tolerogenic DCs, Th2 cells, Treg, and Breg, by promoting NETs formation by neutrophils, and by inducing fetal-protective AAbs in B cells. **(D)** Neutrophils, MØ, MCs, NK cells, and Treg cells are proposed to support uSA remodeling and fetal nourishment. A lack or dysfunctionality of these immune cells may result in improper uSA remodeling followed by fetal undernourishment and fetal growth restriction. AAbs, asymmetric antibodies; ANG-1/2, angiopoietin-1/2; AP, anterior pituitary gland; Breg, regulatory B cell; CCL20, CC-chemokine ligand 20; CT, cytotrophoblast; DC, dendritic cell; PDGF, platelet derived growth factor; FGF, fibroblast growth factor; GAL-1/3, galectin-1/3; GD, glycodelin; IGFBP-1, insulin-like growth factor binding protein-1; H-hCG, hyperglycosylated human chorionic gonadotropin; IL, interleukin; LH, luteinizing hormone; MØ, macrophage; MC, mast cell; M-CSF, macrophage colony stimulating factor; MIF, migration inhibitory factor; MMP-2/9, matrix metalloproteinase-2/9; NETs, neutrophil extracellular traps; NK cell, natural killer cell; p-hCG, pituitary human chorionic gonadotropin; PGF, placental growth factor; PP, posterior pituitary gland; PRL, prolactin; r-hCG, regular human chorionic gonadotropin; ST, syncytiotrophoblast; TGF-β, transforming growth factor-β; TIMPs, tissue inhibitors of matrix metalloproteinases; Treg, regulatory T cell; uSA, uterine spiral artery; VEGF, vascular endothelial growth factor.

Within a few days, after fertilization of the oocyte took place in the fallopian tube, the early embryo in its morula stage enters the uterine cavity. Here, after becoming a blastocyst, the embryo starts to implant into the maternal endometrium (Figure 1). The implantation process is initiated by an apposition reaction followed by the adhesion of the trophoblast cells of the blastocyst to the epithelial layer of the endometrium. Subsequently, trophoblast cells begin to proliferate, differentiate, cross the epithelial basement membrane and invade into the endometrial stroma to form the placenta (1). Uterine spiral arteries (uSA) located within the stroma are targeted by invasive trophoblast cells that replace the endothelial cell layer and provoke alterations in extracellular matrix proteins. Consequently, maternal SA are remodeled from high-resistance into low-resistance vessels with the aim to guarantee a high blood flow from the mother to the fetus by the mid-second trimester and ascertain the fetus to be supplied with sufficient nutrients for an adequate fetal development (2). Impairments in placentation due to shallow trophoblast invasion is associated with late-onset adverse pregnancy outcomes such as intrauterine growth restriction (IUGR) and early-onset preeclampsia (PE) (3) (Figure 1). Moreover, on the maternal side, endometrial stromal cells differentiate into a specialized cell type called decidua cells, via a process called decidualization (4). This structural and functional remodeling of the uterine bed is strongly mediated by the steroid hormones progesterone (P4) and estrogen (E2) (5). In addition, a variety of growth factors, cytokines, prostaglandins, matrix degrading enzymes and their inhibitors as well as adhesion molecules orchestrate the fetal-maternal dialogue and ensure a timely well-defined progression of the highly complex implantation process (6). However, embryo implantation and placentation do not only depend on the availability of individual molecules but also rely on the presence of distinct immune cell populations. These immune cell populations reside in the decidual tissue and are often highly specialized compared to their peripheral counterparts. Unique phenotypic and functional features allow them not only to tolerate the foreign paternal antigens expressed by the fetal tissue but also to actively participate in the different steps of the implantation procedure. Immunological dysregulations are often made responsible for cases of idiopathic infertility and miscarriage underlying the meaning of the maternal immune system for healthy pregnancy progression.

This review will discuss the state-of-the art for the involvement of innate and adaptive immune cell populations in embryo implantation and placentation and will particularly emphasize the role of the hormone human chorionic gonadotropin (hCG) as immune-modulating factor in these processes.

## hCG in Embryo Implantation and Placentation

hCG represents one of the first molecular messages send out by the pre-implanting embryo to modulate the implantation site and to ensure a timely initiation of the nidation process. Despite *CGB* gene expression was proven already in the 8-cell stage embryo (7), active secretion of the hormone starts at the blastocyst stage (8) and enables hCG detection in the maternal circulation 10 days after fertilization. Later on, hCG is produced in high amounts by trophoblast cells (9) resulting in the highest hCG values between the 10th and 11th week of pregnancy. By the end of the first trimester, hCG levels decrease but remain elevated compared to non-pregnant individuals. Notably, a drop of hCG seems to be required for normal pregnancy progression. A recent meta-analysis provided evidence that elevated hCG levels can be detected already at the end of the first trimester in women developing preterm PE (10) and hCG was suggested as a useful predictor for the development and severity of PE (11, 12).

Five different hCG isoforms have been described so far: regular hCG (r-hCG), free-β hCG (hCGβ), hyperglycosylated hCG (H-hCG), hyperglycosylated free-β hCG (H-hCGβ), and pituitary hCG (p-hCG) (13), all of them with distinct biological functions.

r-hCG, produced by syncytiotrophoblast cells is best known for its function to rescue the *corpus luteum* and to maintain P4 production during early pregnancy (14). However, although often neglected, r-hCG has a broader influence on fetal and maternal pathways allowing proper implantation and placentation. This includes the fusion of cytotrophoblast cells into the multinuclear structure of the syncytiotrophoblast (15), the formation of the umbilical circulation in villous tissue and the formation of the umbilical cord (16, 17), the growth of fetal organs (18), the contribution to angiogenesis by forcing the development and growth of uSA (19–21) and the suppression of myometrial contractions (22). Thereby, hCG targets several molecules that are involved in decidualization, implantation, vascularization and tissue remodeling such as prolactin, insulin-like growth factor binding protein-1, macrophage colony stimulating factor, leukemia inhibitory factor (LIF), vascular endothelial growth factor (VEGF), matrix metalloproteinase (MMP)-9, tissue inhibitors of MMPs (TIMPs), galectin-3, and glycodelin (23–26) (Figure 1B).

H-hCG is produced by cytotrophoblast cells and is the most abundant hCG isoform around implantation (27). Its major function is to induce proliferation and invasion of cytotrophoblast cells and it has been reported that H-hCG proportions higher than 50% of total hCG are required for successful embryo implantation (28) (Figure 1B). Whereas, tissue growth factors and collagenases positively modulate H-hCG expression, endothelin-1 and prostaglandin F2α are negative modulators of H-hCG expression (29).

High hCGβ and H-hCGβ levels are also indicative for highly invasive processes as both hCG isoforms support tumor cell growth and survival and their presence is associated with poor prognosis for the patients (30). Finally, p-hCG in collaboration with the luteinizing hormone (LH) promotes ovulation and *corpus luteum* formation during the menstrual cycle (31).

## Clinical Application of hCG in Artificial Reproductive Techniques (ART)—Advantage or Disadvantage?

An increasing number of unintentionally childless couples is seeking help in medical reproduction centers to fulfill their wish of having a child of their own. After several *in vitro* fertilization (IVF)/intracytoplasmic sperm injection (ICSI) cycles using the common clinical protocols after which the patients failed to become or stay pregnant, the demand for unconventional treatment options increases. However, for most of these treatment options there is still no clear evidence for an overall higher success rate or only specific patient groups benefit from these interventions (32). Thus, personalized medicine and the development of new treatment strategies for infertile and miscarriage patients are strongly desired and hCG may represent a promising target in this regard.

hCG is usually applied in two different preparations, either as urine-derived preparation (uhCG) or as recombinant preparation (rhCG) in gonadotropin-releasing hormone agonist or antagonist protocols (33). As a standard procedure, hCG is applied after ovarian stimulation to induce final oocyte maturation. Additionally, some patients receive an intrauterine hCG injection prior to embryo transfer with the aim to improve implantation rates (IR) and live birth rates (LBR). In the majority of recently published studies, uhCG or rhCG is injected into the uterine cavity using an insemination catheter after the cervical mucus is wiped out with a cotton swab or a syringe (34–36). Some study designs also include a flushing step with saline prior to hCG infusion (37). hCG is infused in different doses of 500 up to 1,000 IU solved in either medium or saline and application time points differ between <5 min and days before embryo transfer (38).

As clearly pointed out by Makrigiannakis et al. in their review article in 2017, the efficacy of hCG via the intrauterine hCG application route is controversially discussed (39). Since 2011, several meta-analyses reported different outcomes with regard to IR, clinical pregnancy rates (CPR), ongoing-pregnancy rates (OPR), miscarriage rates (MR), and LBR. The heterogeneity among the different meta-analyses might be attributed to the timing of intrauterine hCG administration meaning the exact time-point of hCG injection prior to embryo transfer as well as the time-point when the embryo is transferred (early cleavage stage or blastocyst stage), the choice of the hCG preparation and the hCG dosage applied. Supportive evidence for improved clinical parameters after intrauterine hCG administration arose from a meta-analysis published by Ye et al. (40) and a potential explanation for the clinical benefit is provided by Strug et al. (41). The latter suggested that hCG counteracts endometrial dyssynchrony resulting from ovarian stimulation and promotes expression of markers essential for the survival of the endometrial stroma (41). On the contrary, Osman et al. (42) and Hou et al. (43) found no beneficial effects on all clinical parameters or showed improvements on only some of them. Notably, Volovsky et al. (44) even demonstrated negative effects on CPR after intrauterine hCG application and this was particularly true for patients without defined repeated implantation failure (RIF).

In a recent Cochrane review from Craciunas et al. (45), it is proposed that in particular patients undergoing cleavage-stage embryo transfer receiving an hCG dosage above 500 IU benefit from intrauterine hCG injection but not patients undergoing blastocyst transfer. The last meta-analysis from 2019 concluded that intrauterine hCG administration before embryo transfer could significantly improve IR, CPR, OPR, and LBR and significantly lowered the MR. These authors suggested that patients treated with 500 IU hCG within 15 min prior to embryo transfer can achieve optimal outcomes (46). Moreover, a recent study by Huang et al. (37) implied that the number of previous implantation failures may influence the efficacy of intrauterine hCG treatment.

Altogether, it can be stated that an overall beneficial effect of intrauterine hCG injection before embryo transfer is not definitely proven and more research has to be done in this field.

## Beneficial Role of hCG in Pregnancy and in ART—Is the Immune System Involved?

There is no doubt that hCG possesses a variety of immune-modulating properties (47, 48). However, how hCG supports immune cells in their function in controlling embryo implantation and placentation is far from being understood. The participation of immune cells in pregnancy-related processes is not restricted to pregnancy itself but begins already during the menstrual cycle before ovulation takes place. Upon the LH surge, an inflammatory reaction is induced where leukocytes are actively recruited to the ovaries through a mechanism involving the leukocyte chemoattractant CCL20. Among these leukocytes are neutrophils, monocytes, natural killer (NK) cells, dendritic cells (DCs), B cells, and T cells and for some of them, a critical role in the ovulation process has been proven (49, 50). Notably, hCG also augments CCL20 expression in the ovaries and thereby stimulates a CCL20-driven leukocyte influx (51). Most likely, this function is attributed to the p-hCG isoform (Figure 1A). Moreover, p-hCG may induce the expression of the macrophage migration inhibitory factor (MIF) in human endometrial stromal cells during the menstrual cycle (Figure 1B). MIF is known to modulate immune responses on his part and to act as a growth and angiogenic factor (52), features that are highly relevant to support remodeling of the endometrial bed.

Later on, during the pre-implantation period local immune cell populations seem to take over critical functions in preparing the implantation site. Approaches where RIF patients received autologous peripheral blood mononuclear cells (PBMCs) before embryo transfer resulted in significantly increased IR and CPR (53, 54). PBMCs were isolated from peripheral blood by using density-gradient centrifugation either on the day of ovulation in fresh embryo transfer cycles or 5 days prior scheduled frozen-thawed embryo transfer cycles. PBMCs were then cultured and monitored for up to 3 days as quality of PBMC cultures was shown to be predictive for the efficiency of PBMC transfers. In each case, 1 × 10^6^ or 4 × 10^7^ PBMCs were transferred into the uterine cavity 2 days before embryo transfer (53, 54).

Markedly, PBMCs that have been activated by hCG prior to their transfer into RIF patients also significantly improved IR, CPR, and LBR irrespective whether the patients underwent fresh or frozen/thawed embryo transfer or whether early cleavage stage embryos or blastocysts were transferred (55–57). Moreover, it became evident that patients with more than three implantation failures may be the group that particularly benefits from this immunotherapy. Several studies were conducted to estimate the underlying mechanisms of an implantation-regulation by hCG-activated PBMCs. One study involved pure mouse material and found that adoptive transfer of PBMCs from non-pregnant mice into mice suffering from an implantation dysfunction elevated the pregnancy rate and increased the endometrial expression of VEGF and LIF during the implantation window (58). Another study combined human PBMCs and mouse embryos. The authors showed that PBMCs from early pregnant women enhanced spreading and invasion of mouse embryos to a greater extent than PBMCs from non-pregnant women. Interestingly, when PBMCs from non-pregnant women were previously exposed to hCG, they possessed a higher capability to promote embryo outgrowth compared to untreated PBMCs (59). Additionally, two studies focusing on human sample material depicted that PBMCs obtained from early pregnant women increased invasion of human trophoblast cells *in vitro*, while PBMCs from non-pregnant women did not. After PBMCs from non-pregnant women were treated with hCG they showed comparable effects on human trophoblast cells as PBMCs from pregnant women (60, 61) suggesting that previous exposure to hCG is required for PBMC-driven trophoblast invasion. Yu et al. (61) further indicated that hCG-activated PBMCs significantly augmented MMP-2, MMP-9, and VEGF and decreased TIMP-1 and TIMP-2 expression in human trophoblast cells.

These findings let assume that hCG administration to patients undergoing ART will support pregnancy in two steps that involve the maternal immune system. First, hCG injection will provoke an active immigration of immune cells into reproductive tissues and thereby direct those cells to the place of action. Second, hCG will activate immigrated and residual immune cell populations in the pre-implantation phase to support these cells in promoting embryo attachment and invasion as well as in forcing the decidualization process.

## hCG-Mediated Immune Regulation Favoring Embryo Implantation and Placentation

Innate and adaptive immune cell types play a pivotal role in the early and late steps of embryo implantation as well as in placentation. hCG is recognized as a key factor in this immune-mediated regulation. However, it remains to be elucidated which specific immune cell populations are targeted by hCG and how they are regulated.

### Neutrophils

Neutrophils are in the first line of innate defense against pathogens to protect the mother and her unborn child. Their functional repertoire includes: phagocytosis, production of granules with potent proteolytic activity and of microbicidal peptides, synthesis of reactive oxygen species, formation of neutrophil extracellular traps (NETs) (Figure 1C), and secretion of pro-inflammatory cytokines and chemokines. Furthermore, they directly interact with macrophages, DCs, NK cells, B cells, and T cells (62). During pregnancy, NETs formation with microbicidal impact is considered as another defense mechanism to protect fetal tissues from infections (63) and hCG was reported to stimulate this pathway (64). In non-pregnant individuals, neutrophils can be found in the cervix, endometrium and fallopian tubes (65, 66) while in pregnant women neutrophils additionally invade the decidua, placenta, and fetal membranes (67, 68). At insemination neutrophils specifically migrate and accumulate around the uterine epithelium (69). Here, they overtake various critical functions associated with angiogenesis, uSA remodeling and trophoblast invasion (70, 71) (Figure 1D). One of the major molecules involved in neutrophil activity with regard to tissue remodeling processes is MMP-9 (72) (Figure 1C). Moreover, human neutrophils under the influence of P4 and E2 induce a specific subpopulation of regulatory T (Treg) cells with pro-angiogenic properties (73). Whether hCG affects neutrophils in a similar way is not known. However, it was described that low doses of hCG inhibit proliferation and induce apoptosis in human neutrophils (74, 75). By doing so, hCG may control neutrophil function as their excessive activation was proven in adverse pregnancy outcomes such as fetal loss and PE (67, 76).

### Monocytes and Macrophages

After their generation in the bone marrow, monocytes typically circulate in the peripheral bloodstream for some days before they enter into tissues and differentiate into tissue-specific macrophages. Here, macrophages fulfill a plethora of different functions including the removal of dead cells and cell debris as well as the presentation of foreign antigens during inflammatory processes (77). Two major subtypes of macrophages have been described: pro-inflammatory M1 cells and anti-inflammatory M2 cells (78). As embryo implantation is a state of controlled inflammation, M1 cells are the main macrophage subtype during this period. Immediately after fertilization, macrophages are actively recruited into the endometrium, myometrium and the decidua and represent the second most abundant immune cell population (79). They support angiogenesis by secreting pro-angiogenic factors like VEGF-A, fibroblast growth factor, platelet derived growth factor, and angiopoietin-1 and−2 (Figure 1C). Moreover, although not directly affecting trophoblast invasion or smooth muscle cell organization, M1 cells promote uSA remodeling by inducing extracellular matrix breakdown and phagocyte apoptotic vascular smooth muscle cells (80). After implantation, macrophages have to switch into the immunomodulatory M2 phenotype to ensure tolerance toward the increasing levels of foreign fetal antigens. This M1 to M2 cell shift seems to be partially fostered by hCG as indicated in human and mouse experimental settings (81, 82) (Figure 1C). On the other hand, hCG stimulates pro-inflammatory functions in human monocytes and macrophages (83, 84) suggesting that in case of an infection, hCG helps to protect the fetus from being attacked. Notably, human placental macrophages are able to produce high amounts of the pro-inflammatory cytokine IL-1 which in turn stimulates hCG secretion by human trophoblast cells (85) proposing a reciprocal interrelationship between macrophages and hCG during pregnancy.

Equally important are fetal macrophages, called Hofbauer cells that infiltrate the villous stroma and reside close to the fetal capillaries (86). Hofbauer cells represent a M2 phenotype and mainly produce IL-10 and TGF-β (87). They promote placental angiogenesis, villous tree growth and branching and protect the fetus from being immunological rejected (88, 89). Likewise human decidual macrophages (90), Hofbauer cells express the LH/CG receptor (91) allowing them to bind and incorporate hCG. This ability seems of fundamental importance in adjusting hCG levels at the fetal-maternal interface and to prevent an aberrant genital differentiation in early pregnancy (92).

### Mast Cells

The contribution of mast cells (MCs) for pregnancy establishment, maintenance, and termination was longtime controversially discussed (93–96). However, nowadays there is accumulating evidence that MCs are indeed critically involved in pregnancy success and in particular regulate fundamental processes during early pregnancy. MCs are present in human (97) and rodent uteri (98, 99) exhibiting a mixed population of different MC types (97, 100). We showed that female mice devoid of MCs were either completely incapable to implant or implanted but showed insufficient uSA remodeling and abnormal placentation (100) resulting in IUGR (101) (Figure 1D). Our and other data further revealed that factors like VEGF (102), galectin-1 (100), and chymases (103) are involved in MC-mediated activities (Figure 1C). As human and mouse MCs express steroid receptors (97, 104) it is suggested that their functionality is affected by the local hormonal environment. In fact, E2 and P4 upregulate chemokine receptor expression on MCs and promote their immigration into the fetal-maternal interface, induce the production of MC mediators and their release by degranulation (104). However, whether hCG possesses similar effects on MCs is unknown as research activities in this field are very limited.

### Natural Killer Cells

Uterine NK cells display a unique profile that differentiates these cells fundamentally from their peripheral counterparts. A high cytokine and angiogenic secretory profile as well as a poor cytotoxic potential are characteristic for uterine NK cells (105). During the first trimester of pregnancy NK cells represent the most abundant immune cell population in decidual tissue (105). However, whether decidual NK cells are derived from peripheral NK cells that enter the fetal-maternal interface and convert into the decidual phenotype and/or expand from residual NK cells is not finally resolved (106, 107). Recently, it was suggested that under the influence of P4 and IL-15, peripheral NK cells start to proliferate, migrate into the implantation site along chemotactic and hormonal gradients provided by trophoblast and endometrial cells and finally convert into decidual NK cells due to high levels of local TGF-β and IL-11 (106).

hCG seems to influence peripheral and endometrial NK cells in several ways although hCG effects on peripheral NK cells are rather inconclusive. While studies from the 80s showed an inhibitory effect of hCG on human peripheral NK cell activity (108, 109), more recent studies suggested a stimulatory effect of hCG on NK cell activity and number (110). Shirshev et al. proposed that hCG levels representative for the first trimester stimulated the expression of specific miRNAs within peripheral NK cells known as positive regulators of NK cell survival, cytolytic activity and production of pro-inflammatory cytokines (111). Notably, higher KIR2DL4^+^ peripheral NK cell numbers and lower implantation rates have been found in patients undergoing IVF and receiving hCG for final oocyte maturation suggesting that in those patients hCG treatment may compromise pregnancy success (112). By contrast, hCG elevates the number of endometrial NK cells through the mannose receptor and may therefore positively influence embryo implantation (113) (Figure 1C).

In line with macrophages and MCs, uterine NK cells are key regulators of embryo implantation and placentation. In both humans and mice, uterine NK cells control trophoblast invasion, support angiogenesis and contribute to proper uSA remodeling (Figure 1D). More specifically, human uterine NK cells either directly force remodeling of the endometrial vascular bed through secretion of MMPs (114) or indirectly by regulating invasive trophoblast cells that in turn promote vascular transformation (115). Human uterine NK cells also produce and secrete a variety of pro-angiogenic factors including VEGF, placental growth factor, angiopoietin-1, and angiogenin-2 (116) (Figure 1C). In mice, uterine NK cells are subdivided in two major subsets based on the expression *Dolichos biflorus agglutinin* (DBA). Whereas, DBA^−^ uNK cells secrete high amounts of IFN-γ and thereby assist in vascular remodeling (117), DBA^+^ uNK cells predominantly produce pro-angiogenic factors (118). In contrast to human uterine NK cells suggested to either enhance or inhibit trophoblast invasion, mouse uNK cells seem to primarily suppress trophoblast motility (119).

Our own research studies identified the heme catabolizing enzyme heme oxygenase-1 (HO-1) as a regulator of uterine NK cell numbers and functionality. HO-1 deficient female mice showed fewer uterine NK cells and lower expression of uterine NK cell-associated angiogenic factors. Moreover, pregnant HO-1 deficient female mice displayed insufficient remodeled uSAs, IUGR fetuses and gestational hypertension (120, 121). The administration of gaseous carbon monoxide, a HO-1 degradation product, normalized uNK numbers and restored uSA remodeling, suggesting that uNKs cells are responsible for SA remodeling. However, fetal impairments are not only attributable to NK absence as their depletion, although interfering with uSA remodeling, did not result in fetal growth restriction (103). This finding, however, contradicts observations made by other research groups (122, 123). Intriguingly, in our NK cell-deficient mouse model, females increase their number of uterine MCs that can compensate for the effects of NK cells and avert major damage to the fetus (124). Accordingly, pregnant females lacking both innate immune cell populations show markedly impaired uSA remodeling and high vascular resistance resulting in disturbed fetal development and growth restricted neonates (101). This was attributable to the chymase Mcpt5, secreted by both cell types (103). Consequently, mice without Mcpt5^+^ cells present a similar phenotype of impaired uSA remodeling and IUGR but not hypertension (103).

### Dendritic Cells

DCs link the innate and the adaptive immune system and are therefore decisive for the induction of late immune responses. Although they are detectable in the uterine tissue before and during pregnancy, compared to macrophages and NK cells, DCs represent a minor immune cell population. DCs accumulate in the uterine tissue when the embryo establishes its first contacts with the maternal endometrium (125). At this time, uterine DCs are critically involved in endometrial changes that are indispensable for further pregnancy progression. In female mice lacking decidual DCs it has been shown that decidual proliferation and differentiation was markedly impaired. Moreover, these females showed perturbed angiogenesis characterized by reduced vascular expansion and attenuated maturation (125). In agreement, impaired homing of CXCR4^+^ DCs into decidual tissue led to a disorganized vasculature with improper uSA remodeling later on (126). As a result, decidual DC-deficient females were unable to implant (125).

DCs also participate in the ovulation process. In follicular fluid, these cells make up a major fraction of all ovarian immune cells and were proven to possess an anti-inflammatory capacity that likely serves to restrict the ovulatory-associated inflammation (127). Furthermore, ovarian DCs are essential for expansion of the cumulus-oocyte complex, release of the ovum from the ovarian follicle, *corpus luteum* formation and enhanced lymphangiogenesis (49). Gonadotropins including hCG induce immigration of DCs into ovaries, thus supporting DC-mediated ovulation control (49) (Figure 1A).

Additionally to their non-immunological activities during pregnancy, DCs are naturally involved in decisions whether fetal tissues are immunologically tolerated or rejected. Typically, DCs take up antigens in peripheral tissues, undergo maturation, immigrate into lymphoid organs and finally present antigen-derived peptides to T cells. By doing so, DCs efficiently prime T cell responses either with a pro- or an anti-inflammatory profile. As presentation of fetal alloantigens may provoke an overwhelming alloreactive T cell response leading to fetal rejection, phenotype, and functions of DCs are modulated during normal pregnancy to restrict detrimental effects for the fetus. This accounts in particular for decidual DCs that in contrast to their peripheral counterparts possess a tolerant, immune regulatory phenotype characterized by a reduced T cell stimulatory capacity (128) and high expression of IL-10 and TGF-β (129). Decidual DCs are able to induce Treg cells and Th2 responses contributing to an overall anti-inflammatory fetal environment (130) (Figure 1C). Both decidua- and trophoblast-derived factors such as E2, soluble HLA-G, glycoprotein 1a, thymic stromal lymphopoietin and galectin-1 instruct DCs to adopt a tolerogenic phenotype (131–135). Moreover, several human and mouse studies identified hCG as a regulator of DCs during pregnancy, however with contradicting results (131, 136–138). Our own analyses revealed that the application of hCG as well as adoptive transfer of hCG-treated tolerogenic DCs into abortion-prone females significantly reduced peripheral and local frequencies of mature DCs, increased Treg cells and protected fetuses from rejection (139, 140). Notably, tolerogenic DCs have to be present at fecundation to confer protection (140) emphasizing their role at very early pregnancy stages. In humans, we proposed hCG as a factor maintaining mature peripheral DCs, and particular myeloid DCs type 1, at low levels but not as a general regulator of DC frequencies during pregnancy (141, 142).

### B Cells

B cells are one of the two major immune cell types belonging to the adaptive branch of the immune system. Best known for their ability to produce immunoglobulins, B cells also participate in antigen presentation and in cytokine production. Early studies from the 70s and 80s investigated the development of an anti-paternal humoral immune response during murine pregnancy and found that this response was restricted to specific allogeneic mating combinations and only became evident after repeated mating cycles. Bell and Billingham (143) further identified the placenta as the tissue provoking the anti-paternal humoral immune response. Nowadays, the participation of B cells in fundamental pregnancy processes is increasingly becoming the focus and it is to be assumed that these cells are also involved in embryo implantation and placentation. We recently observed that pregnant female mice lacking B cells were more susceptible to lipopolysaccharide meaning their fetuses died at doses compatible with fetuses from B cell-competent female mice. This implies a critical role of B cells in the control of bacterial infections during pregnancy (144). Under steady state conditions, mouse studies from the 80s proposed that B cell deficiency during pregnancy does not affect resorption frequencies, litter sizes and neonatal survival (145–147). Our own previous findings show that fetuses of B cell-deficient female mice were smaller compared to fetuses from B cell-competent female mice already within the first half of pregnancy (144). This implies that a lack of B cells during pregnancy, although not leading to complete fetal loss, compromises fetal growth and may thus affect the health of the progeny in long term.

In humans, a B cell deficit evoked by medical intervention before or during pregnancy resulted in an elevated rate of pregnancy-associated complications such as spontaneous abortions, PE, IUGR, and preterm deliveries (148–150) suggesting that B cells are involved in early pregnancy pathways determining pregnancy outcome. However, the study of phenotypic and functional characteristics of uterine B cell populations is impeded by their scarcity at the fetal-maternal interface (151). Analyses of B cell frequencies during mouse and human pregnancy revealed around 1% of mouse B cells and <5% of human B cells in decidual tissue (152, 153). Furthermore, pregnancy-driven changes in B cell numbers of all developmental stages in bone marrow, blood, spleen and lymph nodes (154–160) may affect local B cell frequencies and if deregulated may cause adverse pregnancy outcomes. Indeed, there is evidence that not only a lack but also an overrepresentation of distinct B cell populations at different gestational ages can harm the fetus. For instance, reduced frequencies of regulatory B (Breg) cells during early pregnancy in humans and mice are associated with spontaneous abortions (161, 162). Breg cells comprise all B cells that negatively regulate immune responses and are able to secrete high amounts of immune suppressive cytokines such as IL-10, IL-35, and TGF-β (Figure 1C). We and other research groups demonstrated that hCG not only suppressed the proliferation of mouse splenic B cells but also induced the generation of mouse and human IL-10-producing Breg cells as well as the production of pregnancy-protective asymmetric antibodies (163, 164) (Figure 1C). By doing so, hCG supports the immune suppressive characteristics of B cells and fosters maternal immune tolerance during early pregnancy.

Yet, there are two sides of the medal. In mice, Breg cells are often reported to be part of the CD19^+^ CD5^+^ B1a cell population (165), however B1a B cells depending on their phenotype seem to play an ambivalent role during pregnancy. We found that mouse peritoneal B1a B cells expressing high levels of plasma cell alloantigen 1 and IL-10 support fetal survival, whereas B1a B cells expressing low levels of both marker molecules can induce fetal rejection (166). Moreover, increased frequencies of human B1a B cells during the third trimester of pregnancy were detected in PE patients and were associated with pathologic elevated hCG levels proposing a rather detrimental effect of hCG in this regard. As B1a B cells possess the capacity to produce autoantibodies against the angiotensin II type 1 receptor they were suggested to promote PE-associated symptoms (12). Likewise, production of autoantibodies by increased numbers of plasma cells present in endometrial lesions of endometriosis patients was associated with infertility (167).

Altogether, physiological elevated hCG levels during early pregnancy assist in fetal tolerance induction by promoting the generation and function of Breg cells, whereas high hCG levels at later pregnancy stages may compromise fetal well-being by enhancing autoreactive B cells. Whether hCG encourages B cells in promoting embryo implantation and placentation remains to be elucidated.

### T Cells

T cells being the second major adaptive immune cell type are more abundant at the fetal-maternal interface than B cells and are much better studied. Approximately 10–20% of all decidual leukocytes are T cells, being CD8^+^ T cells more than CD4^+^ T cells including classical as well as regulatory subsets (168). In mice, CD4^+^ T helper (Th) and CD8^+^ T cells represent up to 2% of decidual leukocytes during early and mid-gestation (169). It is suggested that human decidual T cells are highly differentiated, express a broad range of cytokines and cytotoxic markers and exhibit a unique transcriptional profile characterized by strong expression of genes involved in interferon signaling (170). Moreover, although capable to recognize the foreign trophoblastic antigens, decidual T cells do not attack trophoblast cells but rather support their growth and invasiveness (171). Among all decidual T cells, the best described are pro-inflammatory Th1 and Th17 cells as well as anti-inflammatory Th2 and Foxp3^+^ Treg cells and dysregulations in these Th subsets are indicative for several pregnancy complications (172, 173). Nevertheless, the functional properties of decidual T cells are still poorly defined.

Normal pregnancy progression is the result of timely-regulated local shifts between pro- and anti-inflammatory immune responses allowing implantation and placentation in a pro-inflammatory environment, fetal growth in an anti-inflammatory environment and finally the induction of labor and delivery again under pro-inflammatory conditions (174). hCG was shown to affect Th cells in several ways and it is suggested that the hormone advance the passage from a Th1-dominated into a Th2-dominated local environment at the end of the first trimester. hCG is suggested to impair proliferation and to induce apoptosis of conventional T cells (175–179), thus reducing the number of alloreactive T cells that may harm the fetus. Moreover, hCG helps T cells to adopt a suppressor phenotype and to secrete preferentially immune regulatory cytokines such as IL-10 and TGF-β (139, 175, 180, 181). This is true for both human and mouse T cells as both express highly conserved LH/CG receptor molecules (139, 182) and are susceptible to hCG signaling. hCG signaling in turn was proposed to act through the signaling molecules AKT and ERK (183).

Remarkably, *in vivo* hCG administration into mice and humans could significantly improve pregnancy outcomes. We showed that hCG injection during the peri-implantation period into abortion-prone mice increased Treg cell frequencies and significantly reduced the fetal rejection rate (139). In agreement, IVF patients receiving hCG exhibited significantly elevated peripheral Treg cell levels associated with improved IR and LBR when compared to non-hCG-treated controls (36). Likewise, spontaneous abortion patients showed lower peripheral numbers of Th17 cells and elevated Treg cell frequencies after hCG exposure (184, 185), indicating that hCG is able to correct immunological dysbalances associated with adverse pregnancy outcomes. It is worthy to speculate that hCG not only increases peripheral Treg cells but also augments the local Treg cell pool. By this means, hCG may act through three different pathways: (A) active recruitment of peripheral Treg cells into the fetal-maternal interface (186), (B) local expansion of decidual Treg cells (82), and (C) conversion of conventional T cells into Treg cells (181, 183) (Figure 1C).

Recently, Robertson and colleagues provided novel insights into the functionality of decidual Treg cells. The authors proposed a role of decidual Treg cells in preventing excessive inflammatory responses evoked by local effector T cells, a supportive function for other leukocytes and non-hematopoietic cells in the implantation process and a direct involvement of decidual Treg cells in adaptions of the maternal vascular bed (187) (Figure 1D). All these mechanisms are pivotal for the embryo to successfully pass the first steps of its development and because of their modulation by hCG, it can be stated that this unique hormone is critically involved.

## Concluding Remarks

During early pregnancy, innate and adaptive immune cells participate in several fundamental physiological processes including trophoblast invasion, decidualization, angiogenesis, and placentation, all of them allowing proper fetal development and growth. Hereby, some of the immune cells work in close relationships as suggested for MCs, NK cells, and DCs. Moreover, as emphasized throughout this review, hCG is a molecule with a multitude of immunological properties. This hormone does not only regulate local immune cell numbers but also forces these cells to adopt a unique phenotype with the aim to support and protect the fetus. Taken together, the literature discussed here may at least partially provide an explanation for the success rates after hCG treatments in ART or miscarriage patients.

## Author Contributions

AS and AZ wrote and revised the manuscript.

### Conflict of Interest

The authors declare that the research was conducted in the absence of any commercial or financial relationships that could be construed as a potential conflict of interest.
